# *De novo* transcriptome assembly of the eight major organs of Sacha Inchi (*Plukenetia volubilis*) and the identification of genes involved in α-linolenic acid metabolism

**DOI:** 10.1186/s12864-018-4774-y

**Published:** 2018-05-22

**Authors:** Xiao-Di Hu, Bang-Zhen Pan, Qiantang Fu, Longjian Niu, Mao-Sheng Chen, Zeng-Fu Xu

**Affiliations:** 1 Key Laboratory of Tropical Plant Resources and Sustainable Use, Xishuangbanna Tropical Botanical Garden, Chinese Academy of Sciences, Menglun, Mengla, 666303 Yunnan China; 2 College of Life Sciences, University of Chinese Academy of Sciences, Beijing, 100049 China

**Keywords:** Sacha Inchi, *de novo* transcriptome, Organ-specific gene expression, α-linolenic acid metabolism, *Plukenetia volubilis*

## Abstract

**Background:**

Sacha Inchi (*Plukenetia volubilis* L.), which belongs to the Euphorbiaceae, has been considered a new potential oil crop because of its high content of polyunsaturated fatty acids in its seed oil. The seed oil especially contains high amounts of α-linolenic acid (ALA), which is useful for the prevention of various diseases. However, little is known about the genetic information and genome sequence of Sacha Inchi, which has largely hindered functional genomics and molecular breeding studies.

**Results:**

In this study, a *de novo* transcriptome assembly based on transcripts sequenced in eight major organs, including roots, stems, shoot apexes, mature leaves, male flowers, female flowers, fruits, and seeds of Sacha Inchi was performed, resulting in a set of 124,750 non-redundant putative transcripts having an average length of 851 bp and an N50 value of 1909 bp. Organ-specific unigenes analysis revealed that the most organ-specific transcripts are found in female flowers (2244 unigenes), whereas a relatively small amount of unigenes are detected to be expressed specifically in other organs with the least in stems (24 unigenes). A total of 42,987 simple sequence repeats (SSRs) were detected, which will contribute to the marker assisted selection breeding of Sacha Inchi. We analyzed expression of genes related to the α-linolenic acid metabolism based on the *de novo* assembly and annotation transcriptome in Sacha Inchi. It appears that Sacha Inchi accumulates high level of ALA in seeds by strong expression of biosynthesis-related genes and weak expression of degradation-related genes. In particular, the up-regulation of *FAD3* and *FAD7* is consistent with high level of ALA in seeds of Sacha Inchi compared with in other organs. Meanwhile, several transcription factors (*ABI3*, *LEC1* and *FUS3*) may regulate key genes involved in oil accumulation in seeds of Sacha Inchi.

**Conclusions:**

The transcriptome of major organs of Sacha Inchi has been sequenced and *de novo* assembled, which will expand the genetic information for functional genomic studies of Sacha Inchi. In addition, the identification of candidate genes involved in ALA metabolism will provide useful resources for the genetic improvement of Sacha Inchi and the metabolic engineering of ALA biosynthesis in other plants.

**Electronic supplementary material:**

The online version of this article (10.1186/s12864-018-4774-y) contains supplementary material, which is available to authorized users.

## Background

Sacha Inchi (*Plukenetia volubilis* L.), a member of the Euphorbiaceae family [1], is native to the rainforests of South America [2] . Sacha Inchi is also known as Inca peanut, wild peanut, Sacha peanut or mountain peanut, having been cultivated for centuries by the indigenous population [3]. And thus, the magnitude of several evolutionary forces like selection, genetic drift and gene flow on population structure of Sacha Inchi was decided by strong anthropogenic influence [4]. Based on cytological study, the most common chromosome number is 2n = 58 [5]. Sacha Inchi seeds contain 41–54% oil [2, 3, 6], which is characterized predominantly by high levels of polyunsaturated fatty acids (PUFAs), especially α-linolenic acid (ALA, C18:3 *cis* Δ9, 12, 15, ω-3) and linoleic acid (LA, C18:2 *cis* Δ9, 12, ω-6), which represent approximately 50 and 35% of the total oil, respectively [2, 7, 8]. In addition, Sacha Inchi seeds contain substantial amounts of total tocopherols (137 mg/100 g) [7], which are a class of chemical compounds consisting of various methylated phenols and display strong antioxidant activity [7, 9, 10]. Considerable amounts of phytosterols (75.7–86.2 mg/100 g) and 15 polyphenolic compounds, belonging to phenyl alcohol, flavonoid, seicoridoid, and lignan classes, were positively identified in seeds [7, 11]. Particularly, condensed tannins are the main family of phenolic compounds which might be indicative of potential high antioxidant properties [12]. Leaf extracts were characterized by phenolic compounds, steroids, and/or terpenoids, which resulted in the antioxidant activities in leaves of Sacha Inchi [13]. These results indicate that Sacha Inchi could be considered as an important material for production of antioxidant phenolic compounds and phytosterols. ALA and LA are essential fatty acids [14], which are useful in the prevention of coronary heart disease, hypertension, diabetes, arthritis, high cholesterol, cancer, and inflammatory and autoimmune disorders [15–19]. Serum parameters in rats treated with Sacha Inchi oil indicated lower levels of cholesterol and triglycerides, and higher levels of high density lipoprotein in comparison with the control group [20]. The lipid profiles of patients with hypercholesterolemia who intake seed oil of Sacha Inchi for four months indicated a decreased in the values of total cholesterol and non-esterified fatty acids, and a rise in high density lipoprotein and the insulin levels [21]. Moreover, ALA and LA are also critical for the development of infants during pregnancy and breastfeeding periods [8]. However, the amount of ALA in most human diets is insufficient. The ALA/LA ratio in the Western diet is approximately 1:15, which much lower than the ratio recommended by the WHO (1:2 to 1:6) [15, 22]. The Western diet is very high in ω-6 FAs and low in ω-3 FAs because of the unwise recommendation to substitute ω-6 FAs for saturated fats to lower serum cholesterol concentrations, resulting in the production of products rich in ω-6 and poor in ω-3 FAs [23]. Thus, it is necessary to increase the intake of ALA, which can be extracted from the seeds of some oil plants, such as Sacha Inchi. Triacylglycerol (TAG) is the primary unit of energy storage in eukaryotic cells. Normally, TAGs are derived either from the glycerol-3-phosphate (G3P) pathway (also known as the Kennedy pathway) or the acyl-dihydroxyacetone phosphate (acyl-DHAP) pathway [24, 25]. TAG degradation through oxidation (primarily beta-oxidation) releases FAs [26]. One aim of this study is to analyze the expression of genes involved in the G3P pathway and beta-oxidation, which are major pathways for TAG biosynthesis and degradation in most tissues or organisms.

Thus far, most studies on Sacha Inchi have dealt with plant development and physiology [27–29], the characterization of seed oil [3, 6, 8], in vitro regeneration systems [30], and potential applications in biofuel production [31] and in cosmetic, pharmaceutical, and food industries [7, 32–34]. However, the genetic information and molecular mechanisms underlying ALA metabolism in Sacha Inchi have rarely been studied, especially absence of a genome. Only one transcriptome analysis and the expression of oil biosynthesis genes in Sacha Inchi seeds have been published [35, 36]. In the transcriptome analysis, the developing seeds of two stages from two-year-old Sacha Inchi were sequenced and unigenes that may be involved in *de novo* FA and triacylglycerol biosynthesis were identified [35]. In another study, expression profiles of genes controlling unsaturated fatty acids biosynthesis and oil deposition in developing seeds of Sacha Inchi were investigated by quantitative real-time PCR [35, 36]. However, only a few genes contributing to the high level of ALA have been characterized in Sacha Inchi. Our results provide priority candidates for future research.

In this study, we sequenced and *de novo* assembled the transcriptome of 8 major organs of Sacha Inchi using next-generation sequencing technology. The assembled transcriptome sequences will expand the genetic information for functional genomic studies of Sacha Inchi. In addition, the identification of candidate genes involved in ALA metabolism will provide useful resources for the genetic improvement of Sacha Inchi and the metabolic engineering of ALA metabolism in other plants.

## Results

### Transcriptome sequencing and *de novo* assembly of Sacha Inchi

To comprehensively construct the transcriptome of Sacha Inchi, eight major organs, including roots, stems, shoot apexes, mature leaves, male flowers, female flowers, fruits, and seeds, were sampled for RNA isolation. Distinct cDNA libraries of those organs were constructed and sequenced, resulting in a total of 164 G 150-bp paired-end raw reads. After the removal of adapters, poly-N-containing reads and low-quality sequences from the raw data, approximately 162 G clean reads were retained and used for transcriptome assembly and analysis (Additional file 1: Table S1). The Trinity [37] assembly program, which has been shown to be the best single k-mer assembler for RNA-Seq short reads, was used for *de novo* assembly [38]. As a result, an assembly of 349,951 contigs was established for the Sacha Inchi transcriptome. To reduce redundancy and potential assembly errors, the candidate unigenes that most likely had the longest ORFs (Open Reading Frames) were chosen from the assembly result, and then those transcripts were filtered by their fragments per kilobase per million mapped base pairs of sequenced (FPKM) values less than 0.1. Finally, a set of 124,750 unigenes with an average length of 851 bp and an N50 value of 1909 bp was obtained (Table 1). We have compared our data with the seed transcriptome reported by Wang et al. [35] using BLASTn (E-value≤1e-10). The alignment rate of the reported seed transcriptome to our data is 93.8%, indicating the 6.2% of the seed transcriptome was not detected in our dataset, which probably resulted from the seed transcriptome reported by Wang et al. [35] containing transcripts of seeds at two developmental stages, whereas our dataset containing transcripts of seeds at a single developmental stage. The size distribution of unigenes is shown in Additional file 2: Figure S1a. Principal component analysis (PCA) was conducted using R package, the distance assessment reveals that all three independent biological replicates of each sample have good reproducibility, and the seed showed the most distinctive expression patterns in all tested organs (Additional file 3: Figure S2), which is in accord with the result in *Arabidopsis* [39].

**Table 1:** Summary of the assembly and annotation of the Sacha Inchi transcriptome

	No. of sequences
Assembly	
Total number of unigenes	124,750
Total bases (Mb)	145
Average unigene length (bp)	851
N50 (bp)	1909
Number of unigenes (≥ 500 bp)	54,675
Number of unigenes (≥ 1 kb)	29,591
Annotation	No. of matched unigenes (percentage)
Transcript BLASTx against NR	67,832 (54.37%)
Transcript BLASTx against UniRef90	68,063 (54.56%)
Transcript BLASTx against TAIR10	42,109 (33.75%)
Transcript BLASTx against KOG	55,403 (44.41%)
Transcript BLASTx against SwissProt	43,501 (34.87%)
All annotated transcripts	70,124 (56.23%)
Transcripts identified in all five databases	35,381 (28.36%)

To evaluate and summarize the reliability and quality of the assembly, the clean reads were mapped back to the Trinity-assembled transcriptome. The overall alignment rate was 83.39%, indicating that a high-quality *de novo* assembled transcriptome was obtained.

### Functional annotation of the Sacha Inchi transcriptome

After assembly, the 124,750 non-redundant transcripts were subjected to a BLAST search to predict the gene function against five public databases, NR, TAIR10, UniRef90, KOG and Swiss-Prot, and a 10^− 5^ e-value cut-off value was used [40]. We annotated 67,832 (54.37%) unigenes against the NR database, 68,063 (54.56%) unigenes against the UniRef90 database, 42,109 (33.75%) unigenes against the TAIR10 database, 55,403 (44.41%) unigenes against the KOG database, and 43,501 (34.87%) unigenes against the Swiss-Prot database (Table 1). In total, 70,142 (56.23%) unigenes had at least one homologous match from these databases, whereas 35,381 (28.36%) unigenes had significant BLAST matches to proteins in all of the five databases, as shown in a Venn diagram (Additional file 4: Figure S3). The similarity distribution of the top hits showed that 33.99% of the mapped sequences had similarities higher than 80%, while 62.87% of the hits had similarities ranging from 40 to 80% (Additional file 2: Figure S1b). The E-value distribution had a comparable pattern with 38.47% of the mapped sequences with high homologies (< 1e-50), whereas 61.53% of the homologous sequences ranged between 1e-5 and 1e-50 (Additional file 2: Figure S1c). The species distribution of NR BLAST matches is shown in Additional file 2: Figure S1d. The top-scoring BLASTx hits against the NR protein database showed that the top three species were *Ricinus communis* (29.27%), *Jatropha curcas* (9.46%) and *Malus domestica* (3.20%). We further analyzed the unigenes that showed high similarity with those of the KOG database. Among the 25 categories, the largest group was “General function prediction” (11,069, 19.98%), followed by “Posttranslational modification, protein turnover, chaperones” (5827, 10.52%) and “Signal transduction mechanisms” (5752, 10.38%) (Additional file 5: Figure S4). The categories “Cell motility” (66, 0.12%), “Nuclear structure” (160. 0.29%), “Extracellular structures” (208, 0.38%) and “Coenzyme transport and metabolism” (511, 0.92%) accounted for relatively low proportions (Additional file 5: Figure S4).

### Gene ontology and KEGG pathway analysis of the Sacha Inchi transcriptome

We utilized the best BLASTx hit from the NR database to functionally classify the Sacha Inchi unigenes. The best hits were subjected to Blast2GO [41, 42] to analyze the gene ontology (GO) terms and enzyme commission (EC) numbers. The result showed that 45,319 unigenes were assigned to 240,186 GO term annotations, with an average of 5.3 GO terms for each unigene. A total of 45,319 unigene gene functions were described under three main divisions (biological processes, cellular components and molecular functions). The predominant group in each of the biological processes, cellular components and molecular functions was metabolic process (GO: 0008152), cell (GO: 0005623) and catalytic activity (GO: 0003824), respectively (Additional file 6: Figure S5).

To further understand the biological functions and interactions of transcripts, the unigenes of assembled sequences were assigned by the Kyoto Encyclopedia of Genes and Genomes (KEGG) database. The result showed that a total of 24,678 unigenes were involved in 323 different pathways (see Additional file 7).

### Analysis of organ-specific unigenes

Examination of the unigenes expressed in each organ type revealed that female flowers expressed the most unigenes (16%), while stems and seeds expressed the least number of unigenes (11% each) (Additional file 8: Figure S6a). We designated a gene as the organ-specific with the criteria that the expression value (FPKM) was at least 10 in one organ while less than 1 in other organs. Using the criteria, 495, 24, 249, 390, 195, 2244, 287, and 388 unigenes were specifically found in roots, stems, shoot apexes, mature leaves, male flowers, female flowers, fruits, and seeds, respectively (Additional file 8: Figure S6b). Detailed annotation and FPKM information of organ-specific unigenes are listed in Additional file 9. To evaluate the functional properties of these organ-specific unigenes, GO and KEGG annotation enrichment analyses were carried out. As a result, transcripts were significantly enriched in 7, 18, 10, 18 and 22 GO terms in male flowers, female flowers, roots, seeds and stems, respectively (Additional file 10). However, no significantly enriched GO term was found in fruits or mature leaves. Through KEGG pathway enrichment analysis of female flower, most of those pathways are related to metabolism pathways, including “Amino acid metabolism,” “Biosynthesis of other secondary metabolites,” “Carbohydrate metabolism,” “Energy metabolism,” “Glycan biosynthesis and metabolism,” “Lipid metabolism,” “Metabolism of cofactors and vitamins,” “Metabolism of terpenoids and polyketides” and “Nucleotide metabolism,” followed by pathways related to genetic information processing (Additional file 11: Figure S7), and the genes specially expressed in female flowers were significantly enriched in 17 pathways (Additional file 12). Genes specifically expressed in female flowers were enriched not only in pathways related to sugar, lipid, and amino acid metabolism but also in plant hormone signal transduction and phagosome pathways, which is consistent with the GO analysis(Additional file 11: Figure S7).

### Identification and characterization of genes involved in ALA metabolism

**Fig. 1:**
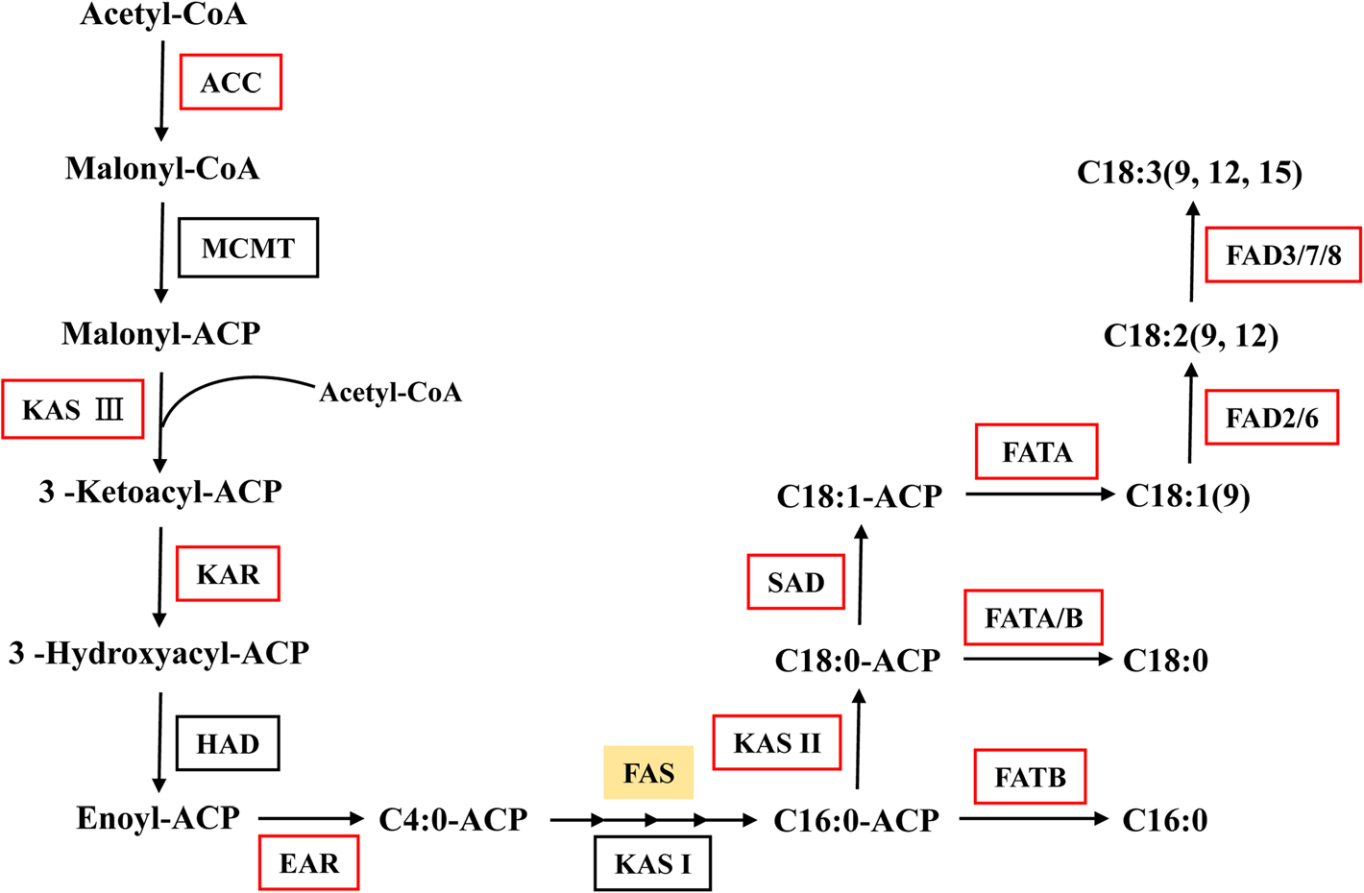
Identification of genes in the pathway of ALA biosynthesis based on the transcriptome of Sacha Inchi. Enzymes involved in ALA biosynthesis are abbreviated as follows: ACC, acetyl-CoA carboxylase; KASI, ketoacyl-ACP Synthase I; KASII, ketoacyl-ACP Synthase II; KASIII, ketoacyl-ACP Synthase III; KAR, ketoacyl-ACP reductase; EAR, enoyl-ACP reductase; FATA, acyl-ACP thioesterase A; FATB, acyl-ACP thioesterase B; SAD, stearoyl-CoA desaturase; FAD2, fatty acid desaturase 2; FAD3, fatty acid desaturase 3; FAD6, fatty acid desaturase 6; FAD7, fatty acid desaturase7; FAD8, fatty acid desaturase 8. Enzymes coded by identified genes are shown in red boxes. This figure was constructed according to the KEGG pathway of ALA metabolism reference pathway (http://aralip.plantbiology.msu.edu/pathways/pathways)

**Fig. 2:**
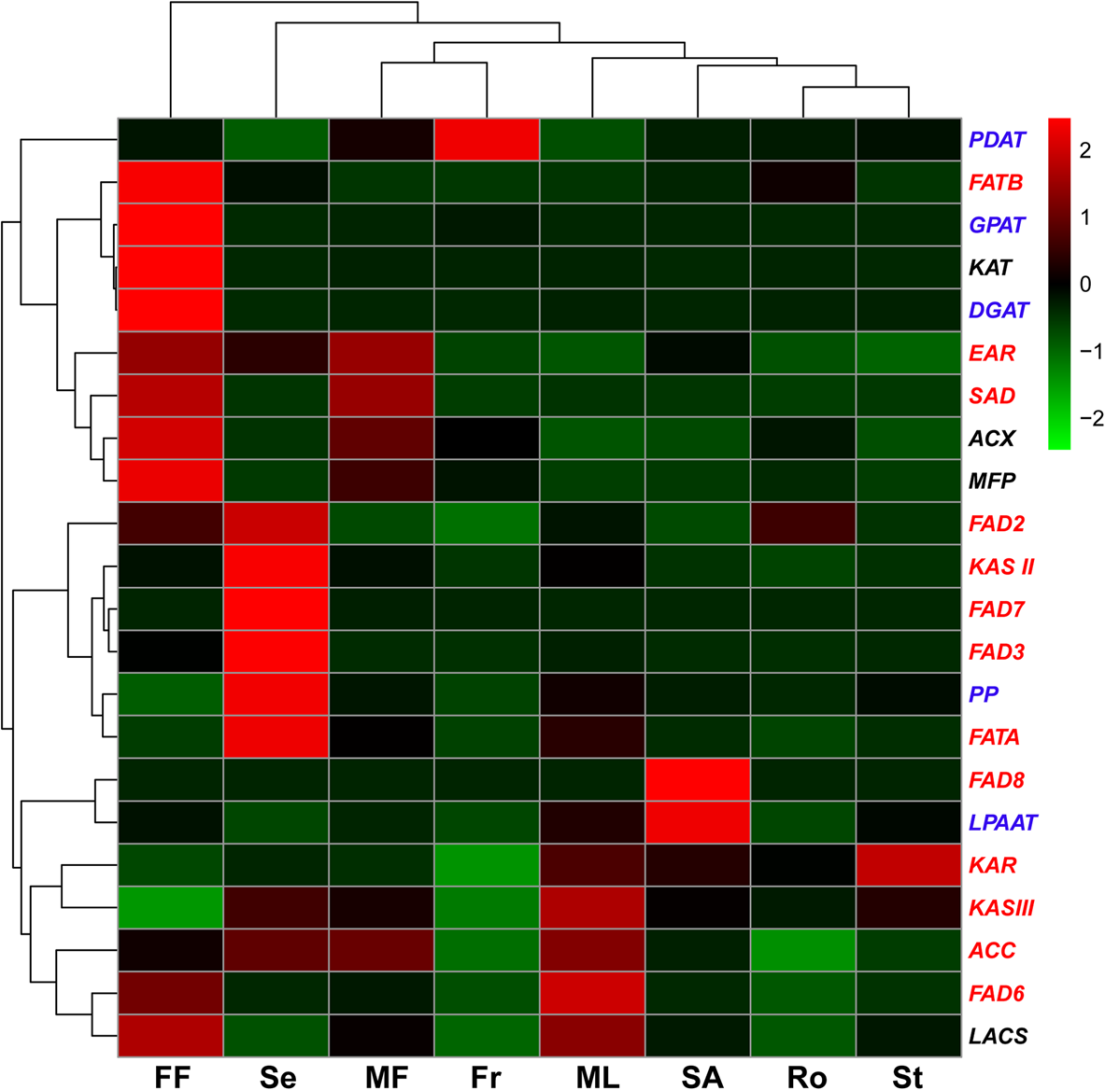
Heat map representation and hierarchical clustering of putative genes involved in ALA metabolism based on the *de novo-*assembled Sacha Inchi transcriptome. FF, female flowers; Fr, fruits; MF, male flowers; ML, mature leaves; Ro, roots; SA, shoot apexes; Se, seeds; St, stems. The genes of the *y*-axis in red are involved in the ALA biosynthesis pathway, those in blue are involved in TAG biosynthesis and those in black are involved in fatty acid catabolism pathway

Based on the *de novo* assembly and annotation of the Sacha Inchi transcriptome, a total of 211 transcripts were identified as candidate unigenes for 13 enzymes of ALA biosynthesis (Table 2 and Additional file 13). The ALA biosynthesis pathway (Fig. 1) is composed of two sub-pathways: fatty acid (FA) elongation and desaturation [43, 44]. The initiation and acyl chain elongation steps of *de novo* FA biosynthesis use acetyl-CoA. Acetyl-CoA is initially catalyzed by acetyl-CoA carboxylase (ACC) to form malonyl-CoA. Then, malonyl-ACP, which is the primary substrate for subsequent elongation, is generated from malonyl-CoA by the malonyl-CoA: ACP malonyl-transferase (MCMT). Four enzymes played a significant role during the addition of two carbons: ketoacyl-ACP synthase III (KAS III), ketoacyl-ACP reductase (KAR), 3-hydroxyacyl-ACP dehydratase (HAD, EC: 4.2.1.-) and enoyl-ACP reductase (EAR) [45, 46]. After four reactions, C4:0-ACP, which is the substrate for further elongation, is produced. Next, ketoacyl-ACP synthase I (KAS I) is used for the elongation from C4 to C16. However, the reaction from C16 to C18 in the ALA biosynthesis pathway is catalyzed by ketoacyl-ACP synthase II (KAS II), and the enzyme stearoyl-ACP desaturase (SAD) removes two hydrogen atoms from stearic acid (18C:0) to form oleic acid (18C:1) during the process of unsaturated FA formation. The enzyme fatty acid desaturase 2 (FAD2) and fatty acid desaturase 6 (FAD6) desaturate oleic acid (C18:1Δ^9^) to generate LA [47]. Fatty acid desaturase 3 (FAD3), FAD7, and FAD8 enzyme genes act on the ω6 fatty acid LA [48, 49] to catalyze the biosynthesis of ALA from LA in Sacha Inchi. In the ALA biosynthesis pathway, the genes encoding FATA, KAS II, FAD2, FAD3, and FAD7 were identified and showed significant up-regulation in seeds compared with other organs (Fig. 2), indicating that the unsaturated FA biosynthesis pathway might be blocked in those organs. However, the genes encoding HAD, MCMT (Malonyl-CoA ACP transferase), and KAS I were not found, which might result from the single development stage of seeds sampled in this study.

**Table 2:** Summary of enzymes involved in ALA metabolism identified by the annotation of the Sacha Inchi transcriptome

ALA biosynthesis			
EC No.	Enzyme abbreviation	Enzyme full name	No. of unigenes
1.1.1.100	KAR	Ketoacyl-ACP reductase	28
1.3.1.9	EAR	Enoyl-ACP reductase	3
1.14.19.1	SAD	Stearoyl-CoA desaturase	66
1.14.19.6	FAD2	Fatty acid desaturase 2	29
1.14.19.22	FAD6	Fatty acid desaturase 6	6
1.14.19.25	FAD3	Fatty acid desaturase 3	8
1.14.19.35	FAD7	Fatty acid desaturase 7	17
1.14.19.36	FAD8	Fatty acid desaturase 8	2
2.3.1.179	KAS II	Ketoacyl-ACP synthase II	19
2.3.1.180	KAS III	Ketoacyl-ACP synthase III	3
3.1.2.14	FATA	Acyl-ACP thioesterase A	2
3.1.2.21	FATB	Acyl-ACP thioesterase B	7
6.4.1.2	ACC	Acetyl-CoA carboxylase	21
Fatty acid catabolism			
EC No.	Enzyme abbreviation	Enzyme full name	No. of unigenes
6.2.1.3	LACS	Long-chain acyl-CoA synthetase	27
4.2.1.17 1.1.1.35	MFP2	Multifunctional protein	15
1.3.3.6	ACX	Acyl-CoA oxidase	16
2.3.1.16	KAT	3-ketoacyl-CoA thiolase	31
TAG biosynthesis			
EC No.	Enzyme abbreviation	Enzyme full name	No. of unigenes
2.3.1.15	GPAT	Glycerol-3-phosphate acyltransferase	12
2.3.1.51	LPAAT	Lysophosphatidic acid acyltransferase	2
3.1.3.4	PP	Phosphatidate phosphatase	10
2.3.1.20	DGAT	Diacylglycerol O-acyltransferase	4
2.3.1.158	PDAT	Phospholipid: diacylglycerol acyltransferase	5

**Fig. 3:**
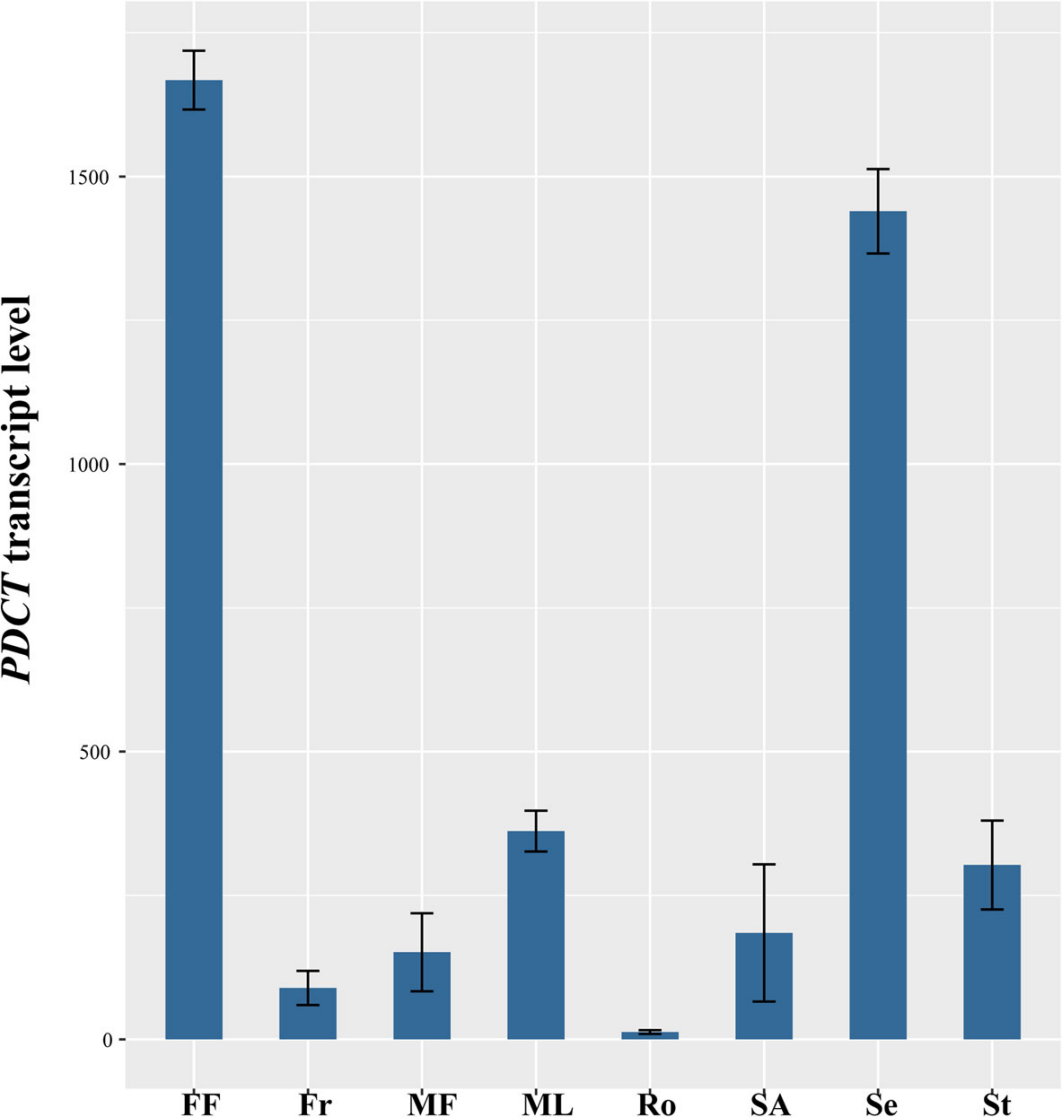
The *PDCT* transcript levels in eight major organs. Values are means ± standard deviations (*n* = 3). FF, female flowers; Fr, fruits; MF, male flowers; ML, mature leaves; Ro, roots; SA, shoot apexes; Se, seeds; St, stems

It is worthy to note that phosphatidylcholine diacylglycerol cholinephosphotransferase (PDCT), which was not found in the previous report of transcriptome of Sacha Inchi seeds [35], but has been shown to play an important role in PUFA accumulation by catalyzing the interconversion between phosphatidylcholine (PC) and diacylglycerol (DAG) [50–52], was found to have high level of expression in female flowers and seeds in this study (Fig. 3).

### Analysis of differentially expressed genes (DEGs) involved in TAG synthesis

**Fig. 4:**
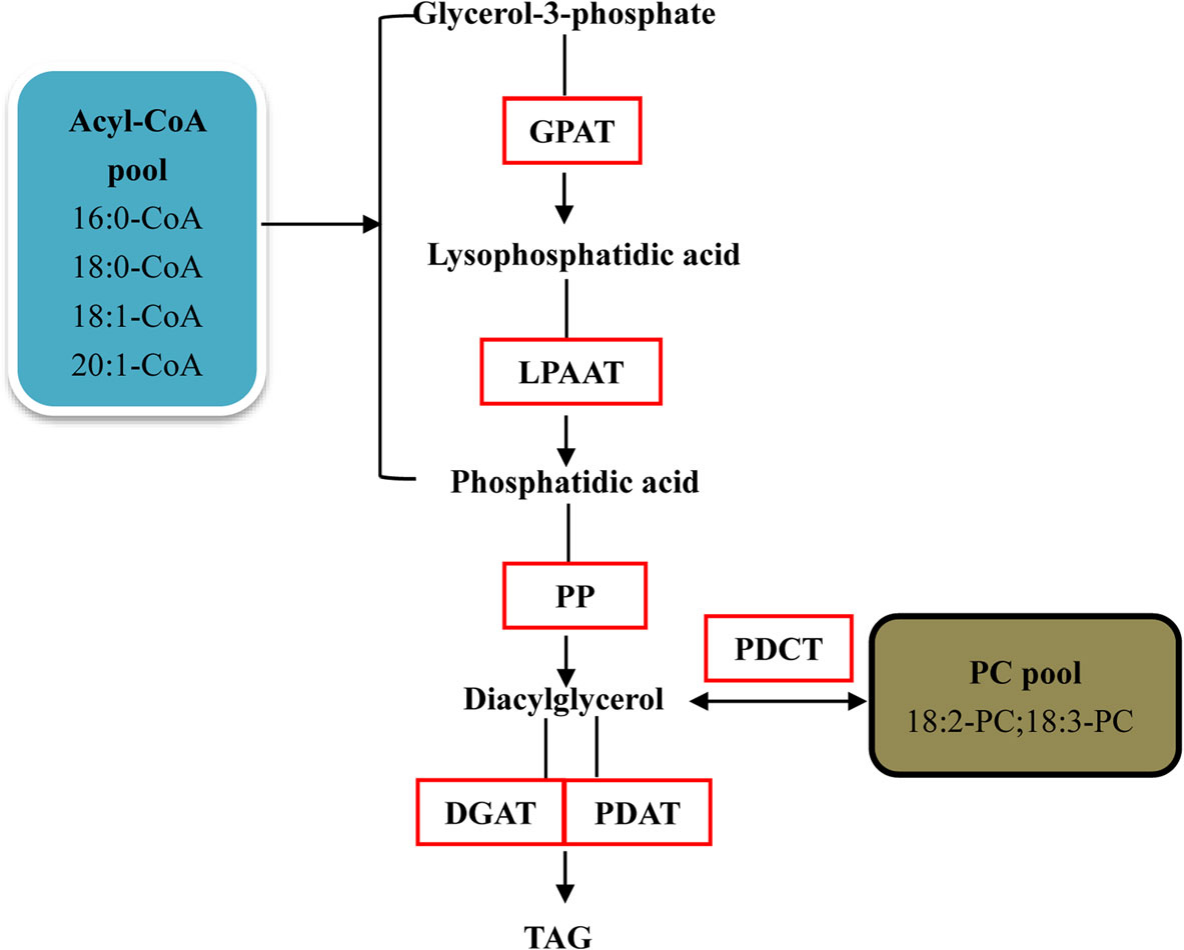
Identification of genes in the pathway of TAG biosynthesis based on the transcriptome of Sacha Inchi. Enzymes are abbreviated as follows:GPAT, Glycerol-3-Phosphate Acyltransferase; LPAAT, Lysophosphatidic acid Acyltransferase; PP, Phosphatidate Phosphatase; DGAT, Diacylglycerol O-Acyltransferase; PDAT, Phospholipid:Diacylglycerol Acyltransferase; PDCT, Phosphatidylcholine Diacylglycerol Cholinephosphotransferase

Differentially expressed genes (DEGs) were analyzed in the eight major organs, especially the genes involved in TAG synthesis, glycolysis/gluconeogenesis pathway (ko00010) and pentose phosphate pathway (ko00030). Genes having a false discovery rate (FDR) value < 0.001 and |log_2_(fold change)| ≥2 found by edgeR were regarded as DEGs [53]. As a result, a total of 24,196 DEGs were detected in our dataset. Among these DEGs, thirty-three genes were involved in TAG biosynthesis (Table 2 and Additional file 13). Glycerol-3-phosphate acyltransferase (GPAT) catalyze the acylation of glycerol-3-phosphate to produce 1-acyl-sn-glycerol-3-phosphate (lysophosphatididic acid, LPA) (Fig. 4) [54, 55]. Phosphatidic acid (PA) is synthesized *de novo* from the acylation of LPA in a reaction catalyzed by acyl-CoA: lysophosphatidic acid acyltransferase (LPAAT). Diacylglycerol (DAG) is produced from PA catalyzed by phosphatidate phosphatase (PP). The DAG is then available for two reactions: diacylglycerol O-acyltransferase (DGAT) transfer acyl-CoAs to the sn-3 position of DAG to produce TAG; alternatively phospholipid: diacylglycerol acyltransferase (PDAT) transfer the sn-2 acyl group from PA to DAG, forming TAG [56, 57]. In addition to the DAG derived from Kennedy pathway, a second PC-derived DAG pool has been identified [58]. The *GPAT* and *DGAT* were up-regulated in female flowers, while *LPAAT*, *PP*, and *PDAT* were up-regulated in shoot apexes, seeds and fruits, respectively (Fig. 2). The glycolysis/gluconeogenesis pathway produces glycerol-3-phosphate which is the source of triacylglycerol (TAG) biosynthesis, and the pentose phosphate pathway is a process of glucose turnover that produces precursor materials and NADPH for FA biosynthesis [54–58]. One hundred and thirty-seven and 64 unigenes were identified as involved in glycolysis/gluconeogenesis pathway and pentose phosphate pathway, respectively, most of which were up-regulated in female flowers and seeds (Additional file 14: Figure S8).

In addition to the key enzymes, we found that some transcription factors related to TAG biosynthesis were highly up-regulated in the seed. *ABSCISIC ACID-INSENSITIVE 3* (*ABI3*), *LEAFY COTYLEDON1* (*LEC1*) and B3-domain transcription factor *FUSCA3* (*FUS3*) showed a 100-fold or more expression difference in seeds compared to other organs (Table 3). These transcription factors probably regulate the seed oil by directly or indirectly regulating FA biosynthesis or TAG accumulation [59].

**Table 3:** Expression levels of transcription factors *PvoABI3*, *PvoLEC1*, and *PvoFUS3* detected in the transcriptome of Sacha Inchi

	Male flowers	Fruits	Mature leaves	Female flowers	Roots	Shoot apexes	Seeds	Stems
*PvoABI3*	0.0083	0.0000	0.0110	0.0393	0.0420	0.0073	59.3290	0.0110
*PvoLEC1*	0.0343	0.0360	0.0300	0.0283	0.0177	0.0000	66.6107	0.0150
*PvoFUS3*	0.0000	0.0000	0.0000	0.0000	0.1130	0.0297	6.8967	0.4457

### Identification and characterization of genes involved in FA catabolism

**Fig. 5:**
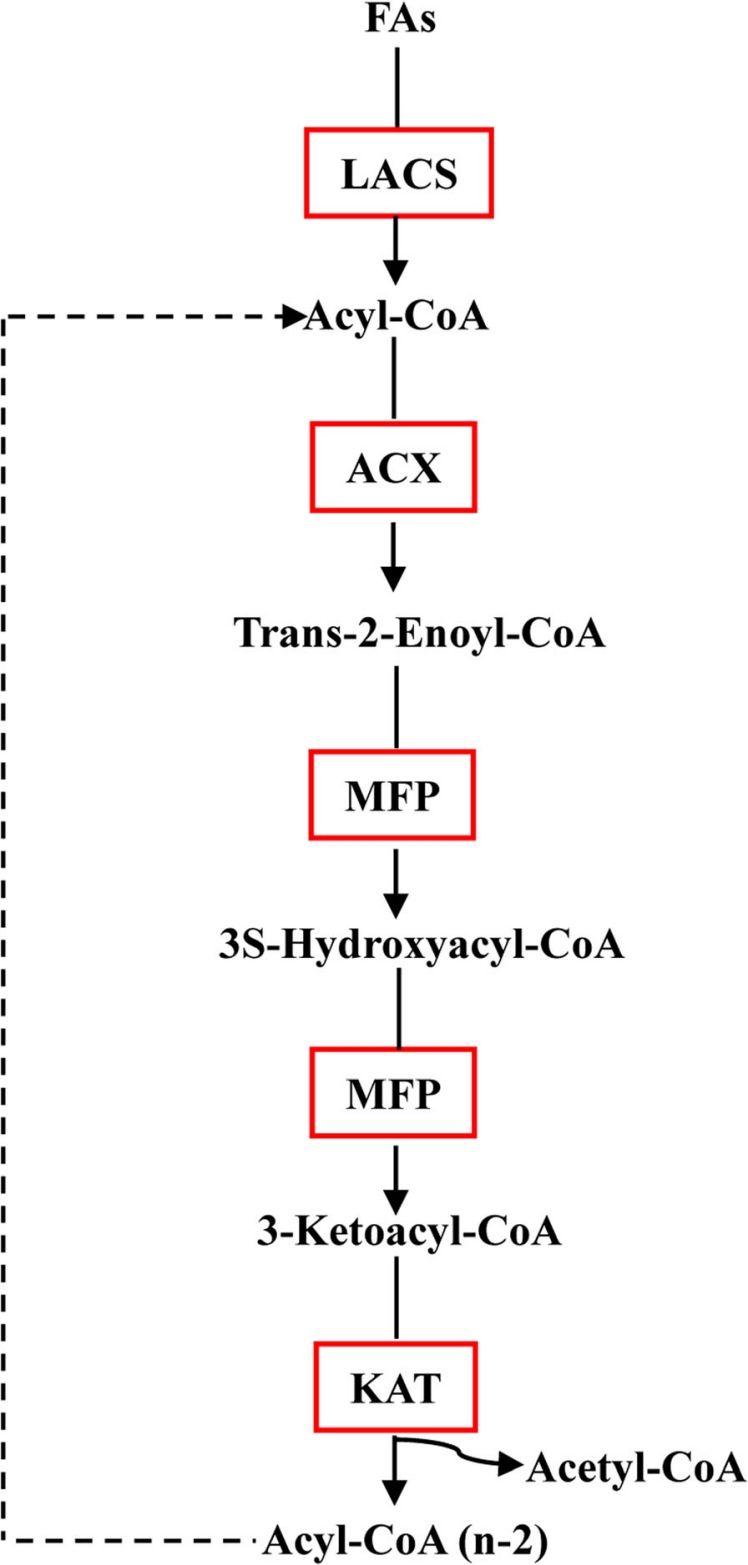
Identification of genes in the fatty acid catabolism pathway based on the transcriptome of Sacha Inchi. Enzymes involved in FA catabolism are abbreviated as follows: LACS, long-chain acyl-CoA synthetase; ACX, acyl-CoA oxidase; MFP, enoyl-CoA hydratase;KAT, 3-ketoacyl-CoA thiolase

The major pathway of FA catabolism, beta-oxidation pathway, is composed of acyl-CoA oxidase (ACX), ketoacyl-CoA thiolase (KAT) and multifunctional protein (MFP) (Fig. 5). Based on the *de novo* assembly and annotation of the Sacha Inchi transcriptome, a total of 89 transcripts were identified as candidate unigenes for 4 enzymes of fatty acid catabolism pathway, including 27 unigenes encoding long-chain acyl-CoA synthetase (LACS) that catalyzes initial reactions of FA catabolism, 16 unigenes encoding ACX, 15 unigenes encoding MFP, and 31 unigenes encoding KAT (Table 2 and Additional file 13). Most of the FA catabolism-related genes exhibited weak expression in seeds, for example, ACX and MFP that catalyze the first and second steps of the β-oxidation of fatty acids, generating acetyl-CoA and energy [60, 61] were down-regulated in seeds (Fig. 2).

### Identification of simple sequence repeats (SSRs)

To develop molecular markers for genetic analysis and marker-assisted selection breeding of Sacha Inchi, simple sequence repeats (SSRs) were identified in our transcriptome. Here, 42,987 SSRs were detected in all of the 124,750 assembled unigenes using MISA software (Additional file 15). Of the SSRs unigenes, 9902 sequences contained more than one SSR and 4788 SSRs were presented in compound formation (Table 4). Of the 42,987 detected SSRs, monomer nucleotide repeats were the most abundant type (30,088; 69.99%), followed by dimer nucleotides (7345; 17.09%), trimer nucleotides (5061; 11.77%), tetramer nucleotides (356; 0.83%), pentamer nucleotides (46; 0.11%) and hexamer nucleotides (91; 0.21%). Among the 42,987 nucleotide repeats, A/T (69.40%) was the most abundant motifs, followed by AT/AT (9.02%) and AG/CT (5.64%).

**Table 4:** Summary of SSR searching results in the Sacha Inchi transcriptome

Item	Number
Total number of sequences examined	124,750
Total size of examined sequences (bp)	106,216,748
Total number of identified SSRs	42,987
Number of SSR containing sequences	27,420
Number of sequences containing more than one SSR	9902
Number of SSRs present in compound formation	4788

### Validation of gene expression profiles using qRT-PCR

**Fig. 6:**
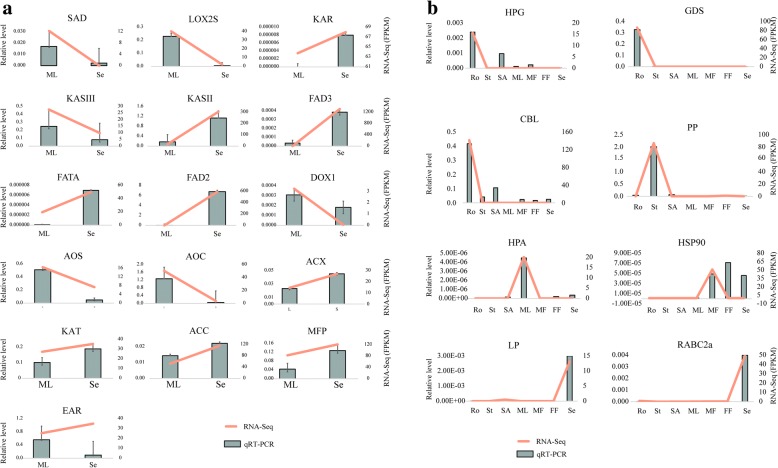
Validation of gene expression profiles by quantitative real-time PCR. **a** Oil-related genes. The full name of genes can been found in the legends of Figs. 1, 4 and 5. Values are means ± standard deviations (*n* = 3). **b** Organ-specific genes. HPG, hypothetical protein GLOINDRAFT_348631; GDS, glutamine-dependent NAD(+) synthetase; CBL, CBL-interacting serine/threonine-protein kinase; PP, peroxidase 64 precursor; HPA, hypothetical protein AALP_AAs53585U000100; HSP90, HSP90 co-chaperone CPR7; LP, phospholipid transfer protein; RABC2a, ras-relatedprotein RABC2a-like. FF, female flowers; Fr, fruits; MF, male flowers; ML, mature leaves; Ro, roots; SA, shoot apexes; Se, seeds; St, stems. The levels of the detected amplicons were normalized using the amplified product of *ACTIN* of Sacha Inchi (*n* = 3)

To experimentally confirm the expression patterns of genes identified by transcriptome sequencing, 9 key unigenes involved in ALA metabolism (Fig. 6a), 7 unigenes detected in our data and 8 unigenes showing organ-specific expression (Fig. 6b) in Sacha Inchi were chosen for qRT-PCR analysis. The detailed information of the selected unigenes is presented in Additional file 16. The results showed that the expression patterns of the most genes tested by qPCR and RNA-Seq were consistent (Fig. 6a, b). Overall, a highly significant correlation (Pearson correlation coefficient *r* = 0.835) existed between qRT-PCR and RNA-Seq results regarding the ratios of gene expression levels (Additional file 17: Figure S9), suggesting that our transcriptome data reflect the expression patterns of most genes in Sacha Inchi.

## Discussion

In this study, we provide a large number of organ-specific unigenes by analyzing the gene expression profile of individual organs. As shown in Additional file 8: Figure S6b, it is apparent that different organs expressed distinct sets of genes. The vast majority of the organ-specific transcripts are found in female flowers, whereas relative small amount of unigenes are predicted to be expressed specifically in other organs. These findings indicate that the metabolisms, such as lipid metabolism, amino acid metabolism in female flowers are active and involve many more specifically expressed genes. This result also provides a large number of candidate specific promoters of female flowers.

Considering ALA has potential applications in food and pharmaceutical industries [7], the main objective of our research was to comprehensively study the ALA metabolism and investigate the molecular basis of this pathway in Sacha Inchi. The omega-3 fatty acid desaturases, especially FAD3, are key enzymes for the formation of ALA from the desaturation of LA [62, 63]. Overexpression of *FAD3* in roots and seeds led to the increase of ALA in a *fad3–2* mutant of *A. thaliana* [64]. Meanwhile, overexpression of *FAD7* increased levels of ALA that led to series of physiological alterations, such as electrolyte leakage and malondialdehyde contents in tomato [65]. Taken together, we suggested that high transcript levels of *FAD3* and *FAD7* is consistent with high level of ALA content in seeds of Sacha Inchi. Our results showed that the predominant genes related to these core pathways are sequence conserved. Furthermore, previous research showed that when the flax *PDCTs* were co-expressed with *FAD2* and *FAD3*, PUFA levels increased in *Saccharomyces cerevisiae* [50]. The *PDCT* from flax are capable of increasing C18-PUFA levels substantially in metabolically engineered yeast and transgenic *A. thaliana* seeds [50]. These data strongly indicate that those genes appear to play an important role in the determination of PUFA content in TAG synthesis. DEGs that directly and indirectly regulate TAG biosynthesis were identified in our data. In addition to a number of genes related to glycolysis/gluconeogenesis pathway and pentose phosphate pathway were up-regulated expression in female flower and seed, the key gene phosphatidate phosphatase (PP) which catalyzes DAG from PA had a high expression level in seed. The transcription factors also regulate the TAG biosynthesis directly or indirectly in plants. Overexpression of maize *LEC1* can increase seed oil as much as 48% [66]. ABI3 and FUS3 are key regulators in phase transition and seed development, maturation and dormancy, and thereby indirectly regulate oil biosynthesis [26]. The genes involved in ALA biosynthesis are regulated by miRNAs as shown in previous study: KASII and KASIII are regulated by the miR159; KAR is regulated by the miR156b, miR156c, miR156g and miR6029; FATA is regulated by miR801, miR298, miR1430 and miR828; FATB is regulated by miR555 and miR113; and SAD is regulated by miR2163 [67].

**Fig. 7:**
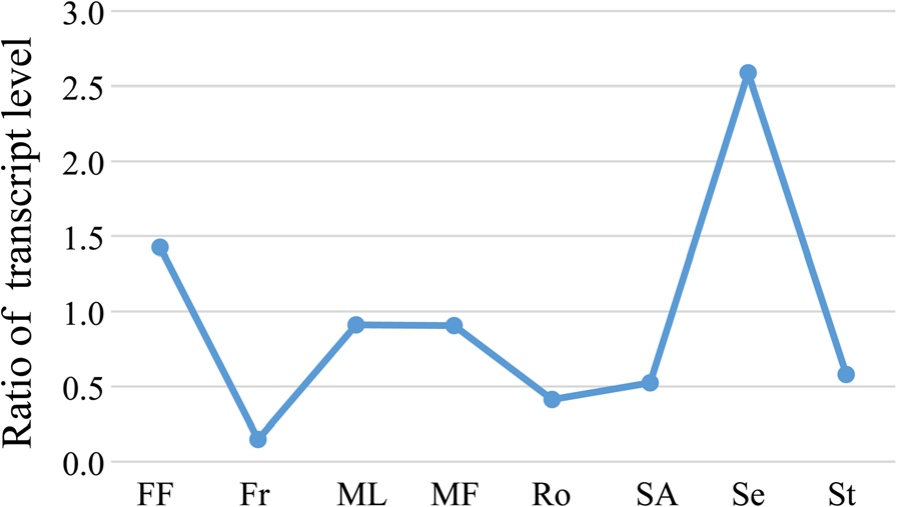
The ratio of expression of genes encoding enzymes for ALA biosynthesis relative to fatty acid catabolism. FF, female flowers; Fr, fruits; MF, male flowers; ML, mature leaves; Ro, roots; SA, shoot apexes; Se, seeds; St, stems

Based on the gene expression pattern analysis, genes coding enzymes related to the ALA biosynthesis strongly express in the seeds, whereas FA catabolism-related genes exhibited weak expression in seeds compared to those in other organs (Figs. 2 and 7),which might related to high ALA content.

## Conclusions

In the present study, the complete transcriptome of Sacha Inchi was *de novo*-assembled and annotated for the first time, generating a total of 124,750 non-redundant transcripts, of which 70,142 could be functionally annotated. Among the eight organs analyzed in this study, the largest number of specifically expressed genes was found in female flowers while the least was found in stems. We identified 211 unigenes and 89 unigenes potentially involved in the ALA biosynthesis and FA catabolism pathways, respectively. Compared with other organs, most of the unigenes related to ALA biosynthesis metabolism were up-regulated, whereas most of those enzymes related to FA catabolism were down-regulated in seeds of Sacha Inchi. In particular, the up-regulation of *FAD3* and *FAD7* may play an important role in high level accumulation of ALA in seeds of Sacha Inchi. Some transcription factors are highly up-regulated in seeds, which are potentially related to TAG accumulation. The transcriptome data reported here provide the foundation for the functional genomics research and genetic improvement of Sacha Inchi.

In conclusion, we present a high-quality transcriptome sequence for the Sacha Inchi. The sequences of genes related to organ-specific and ALA metabolism are obtained based on large-scale transcriptomic data, which will enable further metabolomic and gene functional study. This ALA-rich species was studied to form a more diversified set of ALA to eventually increase storage ALA production and satisfy more human need worldwide.

## Methods

### Plant materials, cDNA library construction and sequencing

Eight organs, including roots (Ro), stems (St), shoot apexes (SA), mature leaves (ML), male flowers (MF), female flowers (FF), fruits (Fr), and seeds (Se), were harvested in August from the 1-year-old plants of Sacha Inchi grown at the Xishuangbanna Tropical Botanical Garden (21^o^54’_N, 101^o^46’_E, 580 m above sea level), Chinese Academy of Sciences, Mengla, Yunnan, China under natural climate conditions. August has a mean temperature of 25.1 °C and mean monthly precipitation greater than 200 mm in Xishuangbanna Tropical Botanical Garden [68]. The samples were collected at 60 days after pollination (DAP) when fruits and seeds have reached full size. Three independent biological replicates of each sample were collected from three individual plants. All samples were frozen immediately in liquid nitrogen and then stored at − 80 °C for RNA extraction. A total amount of 3 μg of RNA per sample was used as input material for RNA sample preparation. Using poly-T oligo-attached magnetic beads, mRNA was purified from total RNA by following the manufacturer’s recommendations (NEB, USA). Then, fragmentation was carried out using divalent cations under elevated temperature. Using these short fragments (about 200 bp) as templates, first-strand cDNA was synthesized using random hexamer primers and MMuLV Reverse Transcriptase (RNase H). Second-strand cDNA synthesis was subsequently performed using DNA polymerase I and RNase H. Then, these cDNA fragments were processed by an end-repair and the ligation of adapters, according to the manufacturer’s protocol (Beckman Coulter, Beverly, USA). The products were purified and enriched with PCR for preparing the final sequencing library. Finally, the library quality was assessed using an Agilent Bioanalyzer 2100 system. A TruSeq SR Cluster Kit v3-cBot-HS (Illumina) was used to perform the clustering of index-coded samples. After cluster generation, the library preparations were sequenced on an Illumina Hi-Seq 4000 platform, and 150-bp paired-end reads were generated at the Novogene Bioinformatics Institute, Beijing, P. R. China.

### Sequence data processing and *de novo* assembly

First, raw reads in fastq format were processed through in-house Perl scripts. Then, the clean data were *de novo* assembled using Trinity software [37], with parameter settings of “-trimmomatic,” which removes reads containing adapters, reads containing poly-N and reads of low quality [69].

### Functional annotation and pathway assignments

Unigenes were aligned by BLASTx to the NCBI nonredundant (NR), Swiss-Prot, TAIR10, UniRef90 and KOG databases with a threshold E-value of 10^− 5^. For each unigene, the best BLASTx hit from the NR database was submitted to the BLAST2GO platform [42, 70], and GO terms were obtained based on annotations between gene names and GO terms. To determine metabolic pathways, the unigenes were also submitted to the online KEGG (Kyoto Encyclopedia of Genes and Genomes) Automatic Annotation Server (KAAS), with single-directional best hit method [71].

### Differential expressed genes (DEGs) analysis

The expression level of each unigene was measured using the fragments per kilobase of transcript sequence per millions base pairs sequenced (FPKM) method. The clean reads were aligned to the assembly using Bowtie [72], and the resulting alignments were used to estimate expression abundances in FPKM [37]. All read counts were normalized to FPKM. Differential expression analysis of the samples with three biological replications was performed using the edgeR [53]. Genes with FDR value < 0.001 and |log2(fold change)| ≥2 calculated by edgeR were regarded differentially expressed.

### Simple sequence repeat (SSR) analysis

The Perl script MISA (MIcroSAtellite identification tool, http://pgrc.ipk-gatersleben.de/misa/misa.html) was used to identify SSRs in Sacha Inchi transcriptome sequences [73].

### Validation by quantitative real-time PCR (qRT-PCR)

Gene-specific primer pairs were designed using Primer Premier 5.0 software, and the amplified PCR products varied from 100 to 250 bp (Additional file 15). qRT-PCR assays were performed as previously described [74]. The reference gene was chosen based on the methods of Niu et al. [75].

## Additional files

Additional file 1: **Table S1.** Overview of the sequencing data of the Sacha Inchi transcriptomes. (XLS 25 kb)

Additional file 2: **Figure S1.** Overview of Sacha Inchi transcriptome assembly and the characteristics of the homology search of unigenes against the NR database by BLAST (cut-off E-value of 1.0E-5). (a) Size distribution of the assembled unigenes. (b) Similarity distribution of the best BLAST hits for each unigene. (c) E-value distributions of the best BLAST hits for each unigene against the NR database. (d) Species distribution of the best BLAST hit for each unigene. (TIF 935 kb)

Additional file 3: **Figure S2.** The Pearson correlation coefficient (r) was used to estimate the difference between the replicates of each tissue. The number between these two samples is given in the plot. The color represents r value, which shows high correlation in red between two samples, while low correlation in blue. (TIF 4328 kb)

Additional file 4: **Figure S3.** Venn diagram showing the BLAST searches of the Sacha Inchi transcriptome against the five public databases. *De novo* unigene sequences were used to search against the following public databases: NR, UniRef90, TAIR10, KOG and Swiss-Prot. The numbers of unigenes that have significant hits against the five databases are shown in each intersection in the Venn diagram. (TIF 574 kb)

Additional file 5: **Figure S4.** Histogram presentation of clusters of orthologous group classification of assembled unigenes. A total of 124,750 unigenes were classified into 25 functional categories. (TIF 816 kb)

Additional file 6: **Figure S5.** Distribution of gene ontology (GO) categories of unigenes for Sacha Inchi. GO functional annotations are summarized into three main categories: biological process, cellular component, and molecular function. The number of unigenes in each category is shown on the *y*-axis. (TIF 1684 kb)

Additional file 7: Pathways identified in the Sacha Inchi transcriptome. Three hundred and fifty-three KEGG pathways identified in Sacha Inchi and the corresponding unigene numbers of each pathway are shown. (XLS 60 kb)

Additional file 8: **Figure S6.** The statistics of unigenes in eight organs. a) The percentage of unigenes expressed in each organ. b) Number of organ-specific unigenes. FF, female flowers; Fr, fruits; MF, male flowers; ML, mature leaves; Ro, roots; SA, shoot apexes; Se, seeds; St, stems. (TIF 2536 kb)

Additional file 9: Detailed FPKM and Annotation information of organ-specific unigenes. (XLS 646 kb)

Additional file 10: The list of GO terms that were significantly enriched in male flowers, female flowers, roots, seeds and stems. Gene ontology (GO) terms were assigned to organ-specific unigenes based on the top hits against the NR database. (XLS 33 kb)

Additional file 11: **Figure S7.** Histogram presentation of the KEGG pathway annotation of female flower (FF)-specific genes. (TIF 1210 kb)

Additional file 12: The list of KEGG pathways were enriched in each organ. (XLS 70 kb)

Additional file 13: List of FA and TAG metabolism-related genes detected in Sacha Inchi transcriptome. A) Unigenes related to ALA biosynthesis. B) Unigenes related to the fatty acid catabolism pathway. C) Unigenes related to TAG biosynthesis. (XLS 69 kb)

Additional file 14: **Figure S8.** Heat map representation and hierarchical clustering of putative genes involved in glycolysis/gluconeogenesis pathway and pentose phosphate pathway. A: glycolysis/gluconeogenesis pathway (ko00010). B: pentose phosphate pathway (ko00030). (TIF 2539 kb)

Additional file 15: Overview of the SSRs detected in the assembled unigenes of Sacha Inchi. (XLS 4872 kb)

Additional file 16: Primers for qRT-PCR in this study. (XLS 31 kb)

Additional file 17: **Figure S9.** Pearson correlation analysis of the gene expression ratios obtained from RNA-Seq and qPCR data. The qPCR log_10_ values (expression ratios; *y*-axis) were plotted against the RNA-Seq log_10_ values (*x*-axis). The Pearson correlation coefficient (r) is given in the plot, and the circle indicates the extremely significant difference at *p* < 0.01. (TIF 337 kb)

## Abbreviations

ACC: Acetyl-CoA carboxylase
ACX: Acyl-CoA oxidase
ALA: α-linolenic acid
DAG: Diacylglycerol
DEGs: Differentially expressed genes
DGAT: Diacylglycerol O-acyltransferase
EAR: Enoyl-ACP reductase
EC: Enzyme commission
FAD2: Fatty acid desaturase 2
FAD3: Fatty acid desaturase 3
FAD6: Fatty acid desaturase 6
FAD7: Fatty acid desaturase 7
FAD8: Fatty acid desaturase 8
FATA: Acyl-ACP thioesterase A
FATB: Acyl-ACP thioesterase B
FPKM: Fragments per kilobase per million mapped base pairs of sequenced
GO: Gene ontology
GPAT: Glycerol-3-phosphate acyltransferase
KAR: Ketoacyl-ACP reductase
KASII: Ketoacyl-ACP synthase II
KASIII: Ketoacyl-ACP synthase III
KAT: 3-ketoacyl-CoA thiolase
KEGG: Kyoto Encyclopedia of Genes and Genomes
LA: Linoleic acid
LPAAT: Lysophosphatidic acid acyltransferase
NCBI: National Center for Biotechnology Information
NR: nonredundant
PC: Phosphatidylcholine
PDAT: Phospholipid: diacylglycerol acyltransferase
PDCT: Phosphatidylcholine diacylglycerol cholinephosphotransferase
PP: Phosphatidate phosphatase
PUFAs: Polyunsaturated fatty acids
SAD: Stearoyl-CoA desaturase
TAG: Triacylglycerol
