# Supplementary figures and images for: De novo transcriptome assembly of the eight major organs of Sacha Inchi (Plukenetia volubilis) and the identification of genes involved in α-linolenic acid metabolism
Source: BMC Genomics. 2018 May 22;19:380. doi: 10.1186/s12864-018-4774-y (PMC5964912; doi:10.1186/s12864-018-4774-y)

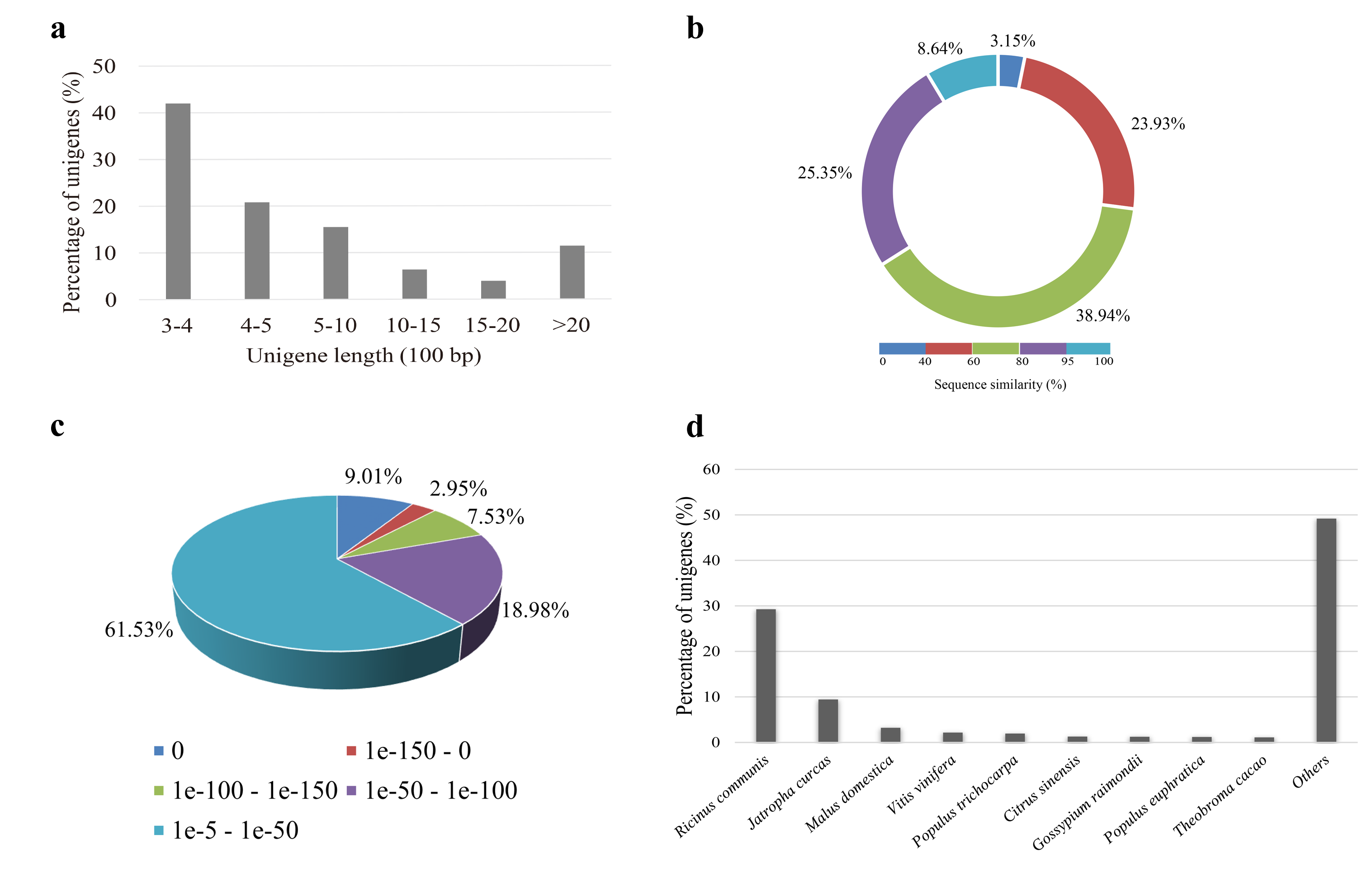

Supplement: Supplementary file 2 — Figure S1. Overview of Sacha Inchi transcriptome assembly and the characteristics of the homology search of unigenes against the NR database by BLAST (cut-off E-value of 1.0E-5). (a) Size distribution of the assembled unigenes. (b) Similarity distribution of the best BLAST hits for each unigene. (c) E-value distributions of the best BLAST hits for each unigene against the NR database. (d) Species distribution of the best BLAST hit for each unigene. (TIF 935 kb) [file 12864_2018_4774_MOESM2_ESM.tif]

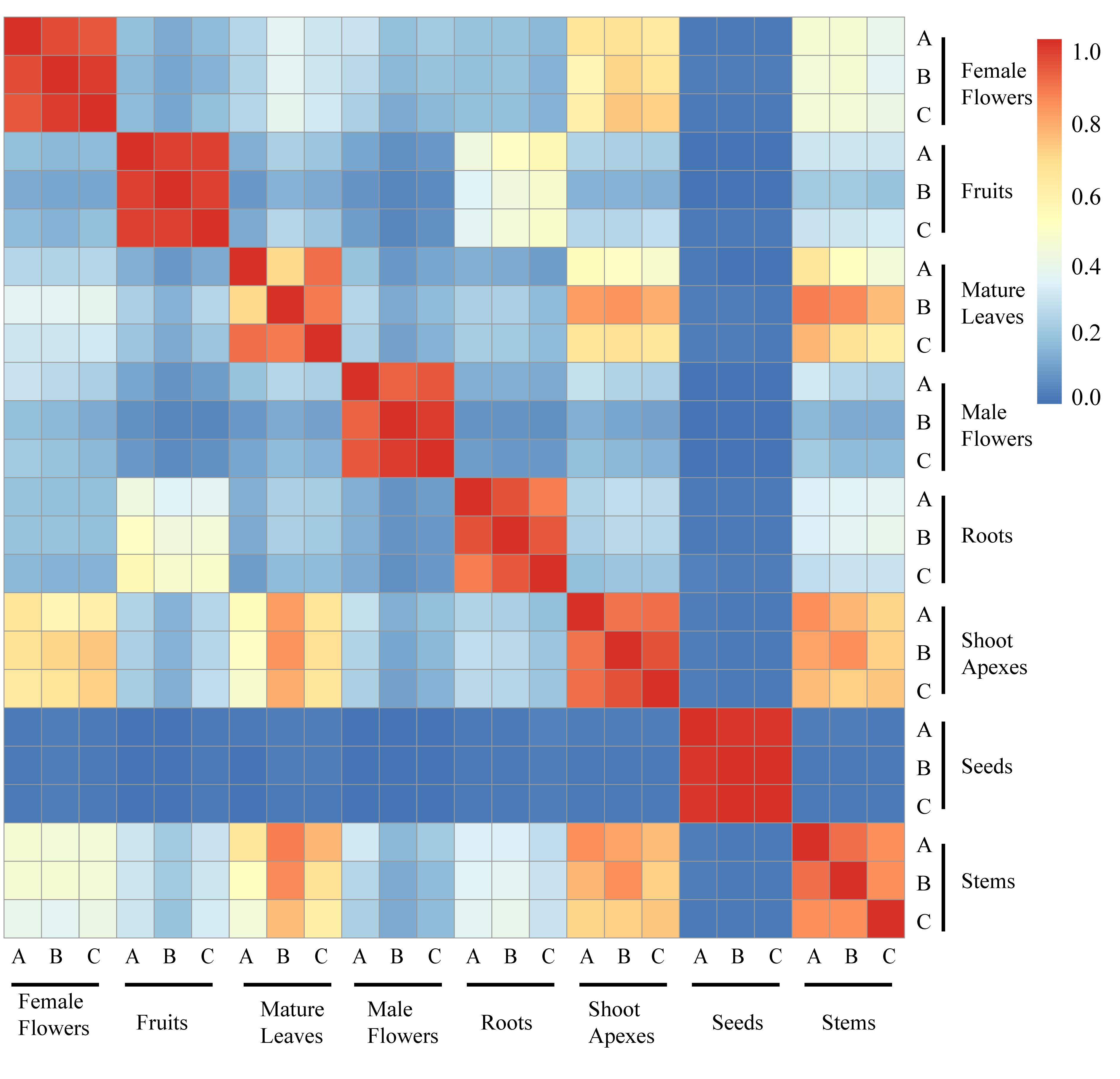

Supplement: Supplementary file 3 — Figure S2. The Pearson correlation coefficient (r) was used to estimate the difference between the replicates of each tissue. The number between these two samples is given in the plot. The color represents r value, which shows high correlation in red between two samples, while low correlation in blue. (TIF 4328 kb) [file 12864_2018_4774_MOESM3_ESM.tif]

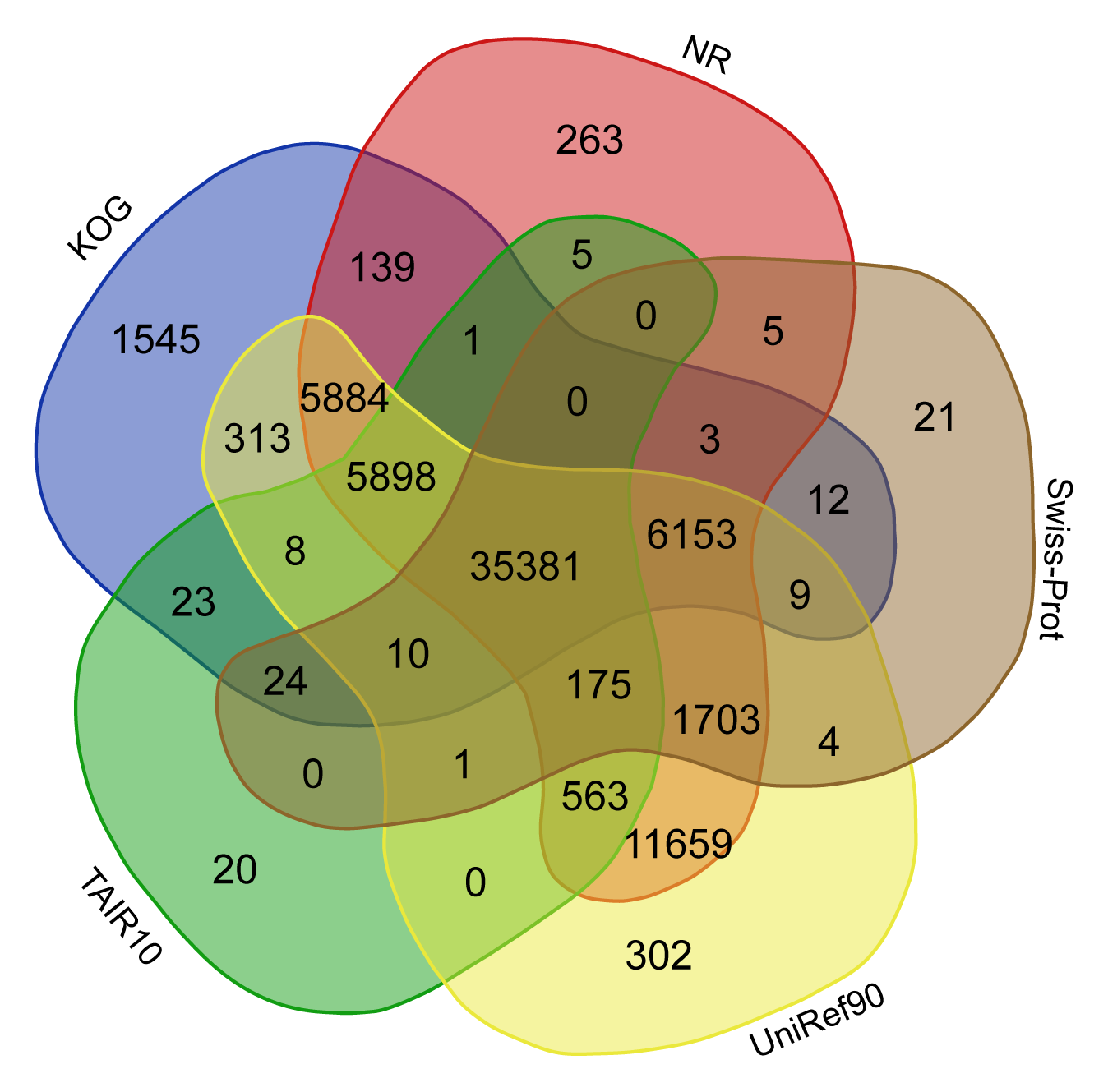

Supplement: Supplementary file 4 — Figure S3. Venn diagram showing the BLAST searches of the Sacha Inchi transcriptome against the five public databases. De novo unigene sequences were used to search against the following public databases: NR, UniRef90, TAIR10, KOG and Swiss-Prot. The numbers of unigenes that have significant hits against the five databases are shown in each intersection in the Venn diagram. (TIF 574 kb) [file 12864_2018_4774_MOESM4_ESM.tif]

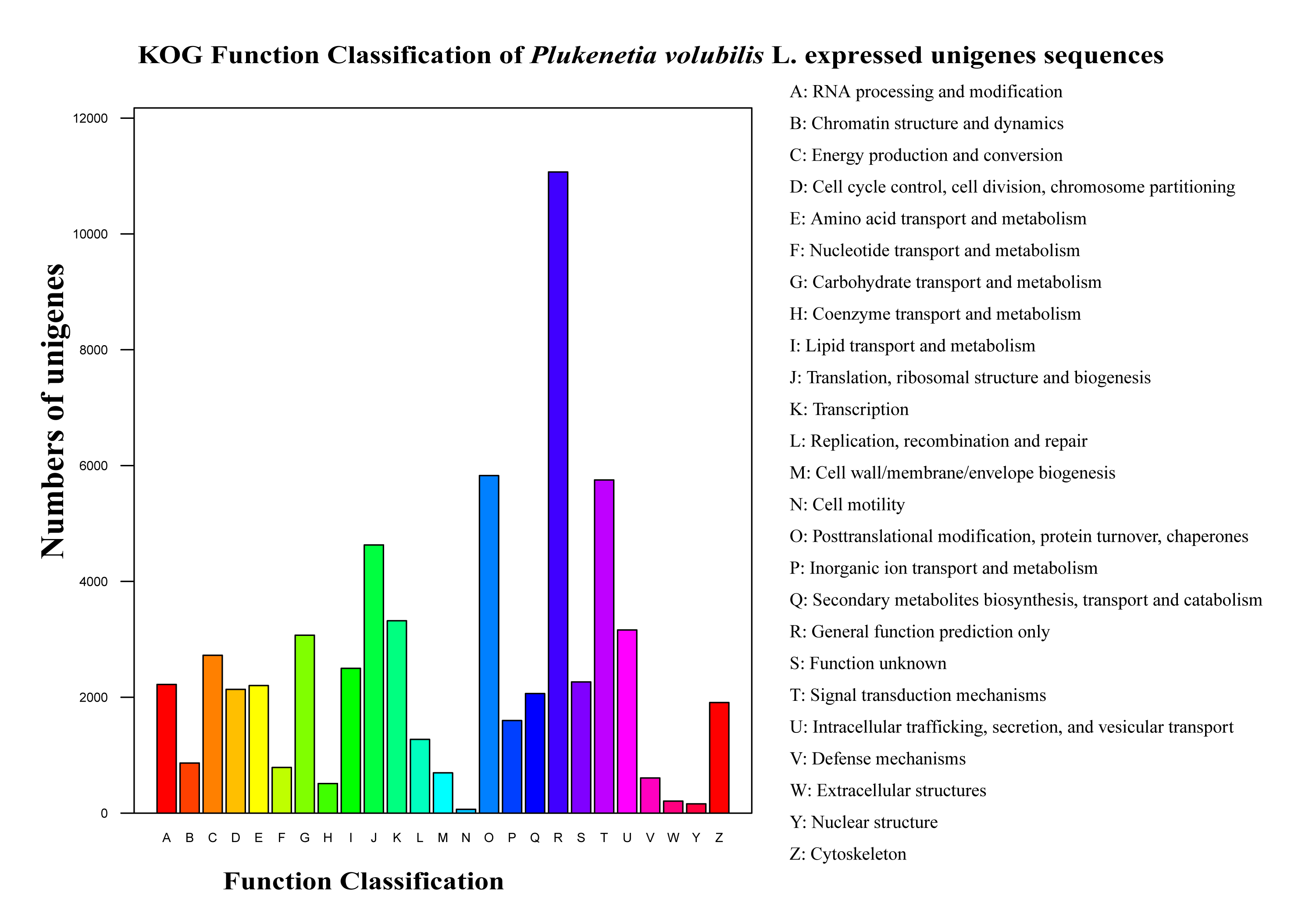

Supplement: Supplementary file 5 — Figure S4. Histogram presentation of clusters of orthologous group classification of assembled unigenes. A total of 124,750 unigenes were classified into 25 functional categories. (TIF 816 kb) [file 12864_2018_4774_MOESM5_ESM.tif]

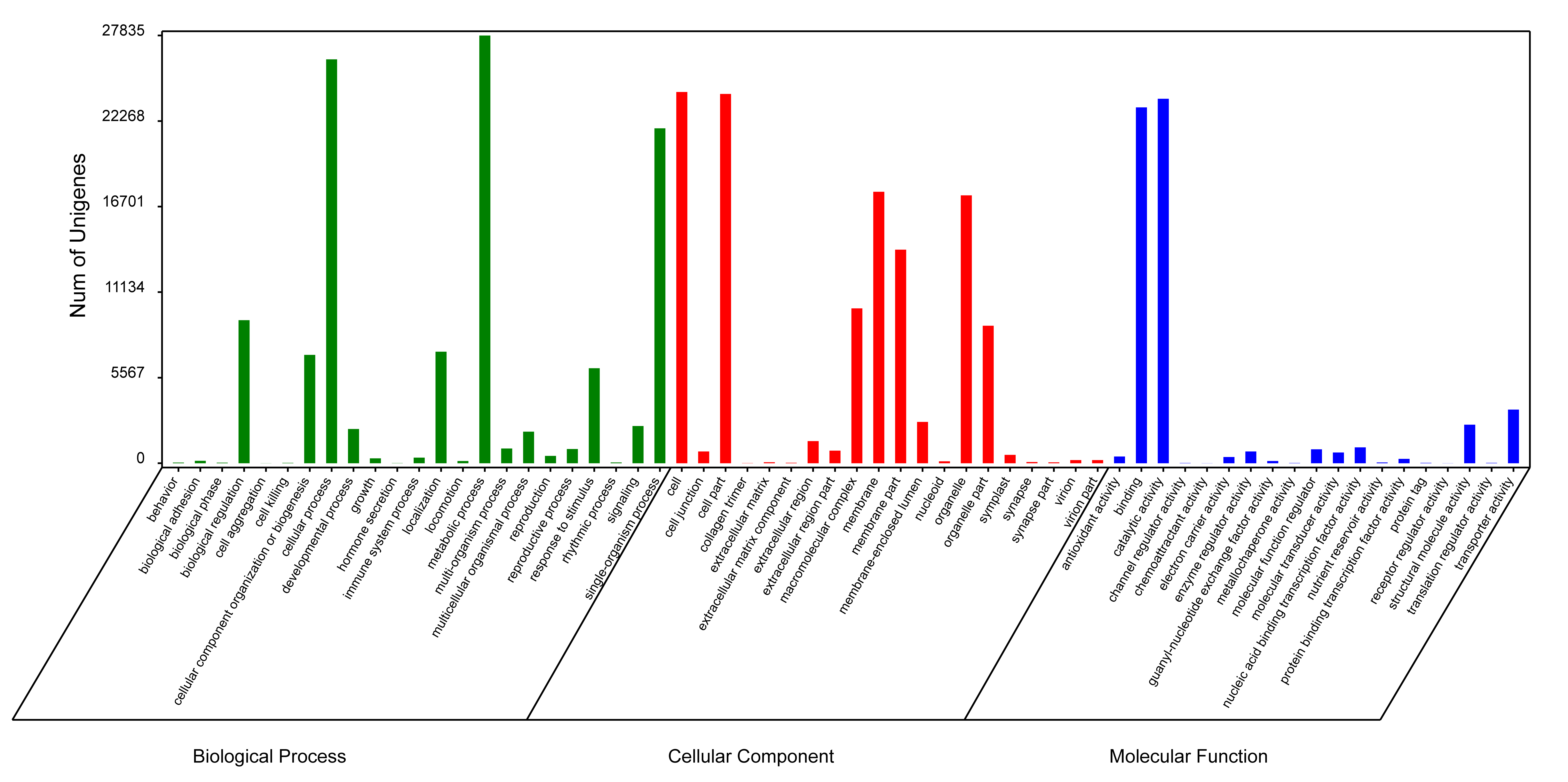

Supplement: Supplementary file 6 — Figure S5. Distribution of gene ontology (GO) categories of unigenes for Sacha Inchi. GO functional annotations are summarized into three main categories: biological process, cellular component, and molecular function. The number of unigenes in each category is shown on the y-axis. (TIF 1684 kb) [file 12864_2018_4774_MOESM6_ESM.tif]

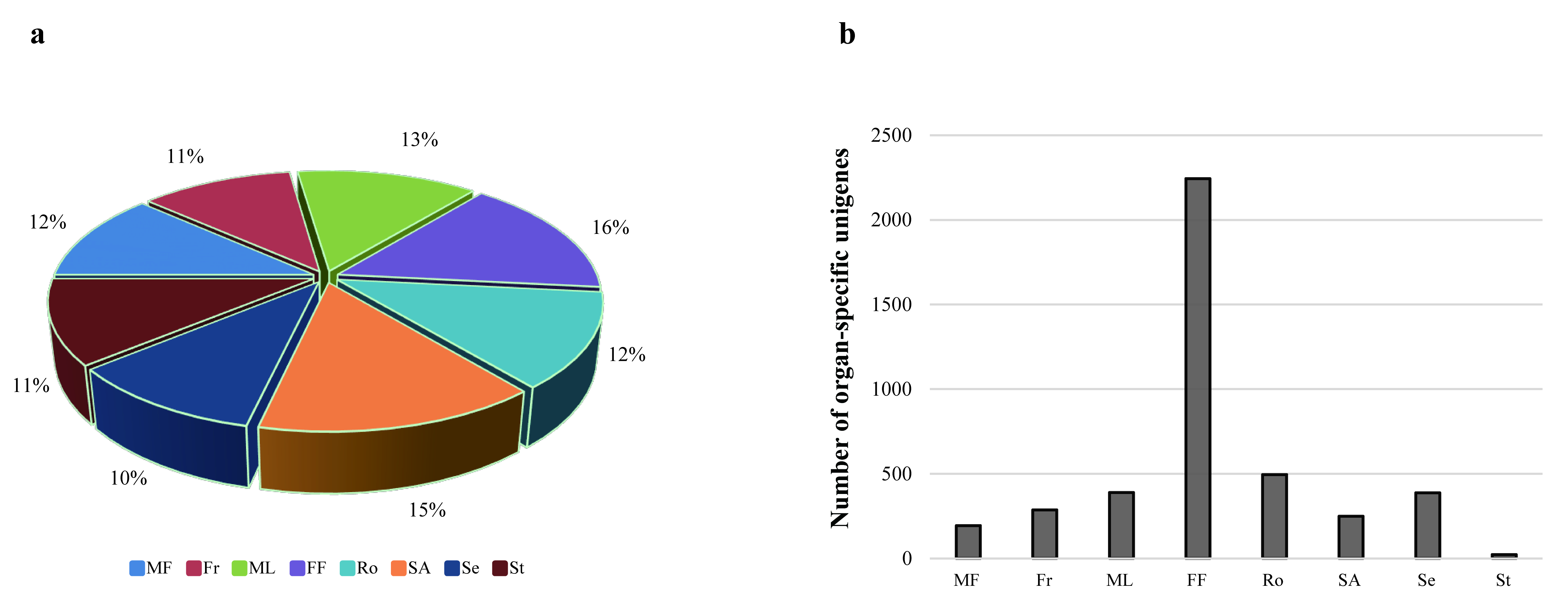

Supplement: Supplementary file 8 — Figure S6. The statistics of unigenes in eight organs. a) The percentage of unigenes expressed in each organ. b) Number of organ-specific unigenes. FF, female flowers; Fr, fruits; MF, male flowers; ML, mature leaves; Ro, roots; SA, shoot apexes; Se, seeds; St, stems. (TIF 2536 kb) [file 12864_2018_4774_MOESM8_ESM.tif]

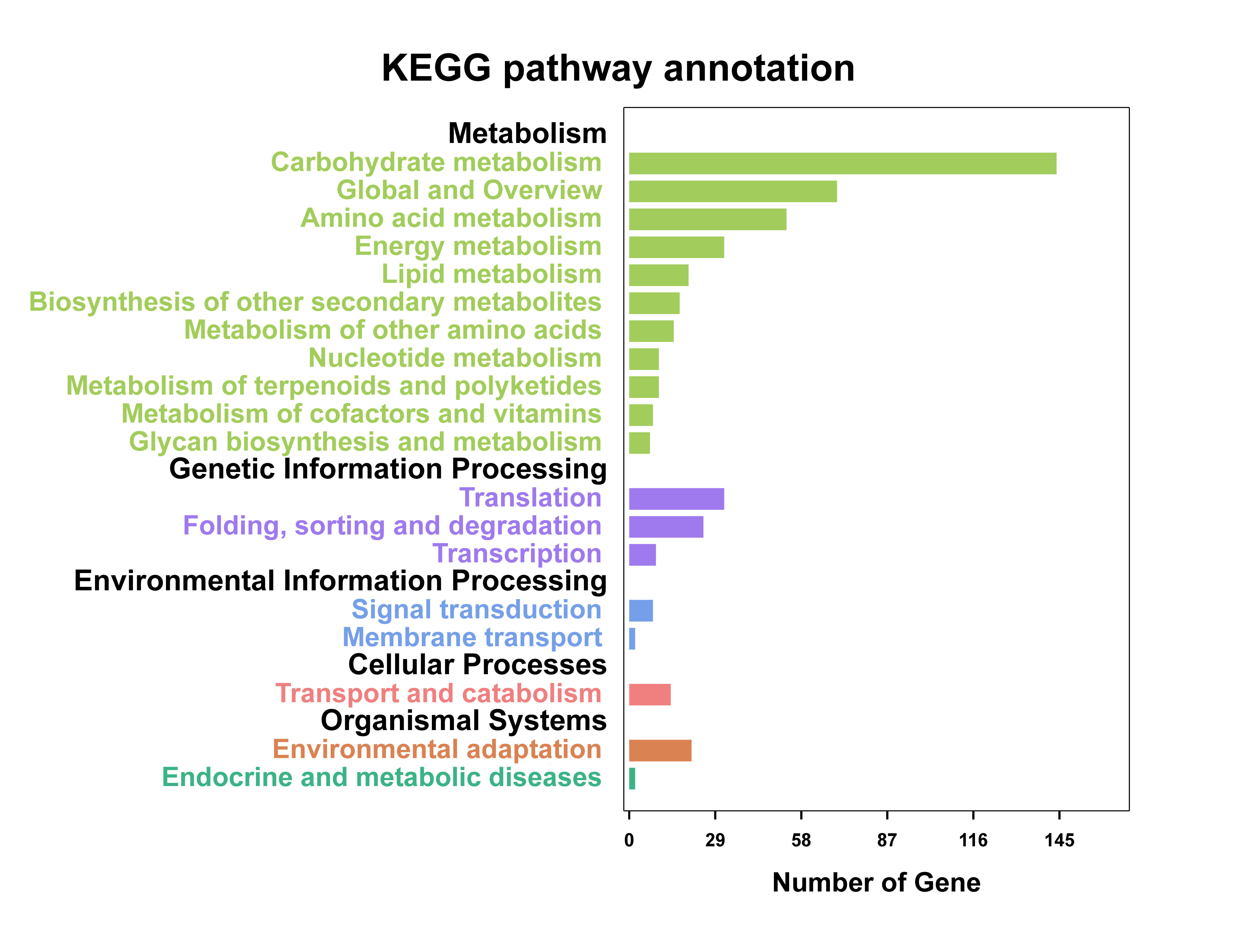

Supplement: Supplementary file 11 — Figure S7. Histogram presentation of the KEGG pathway annotation of female flower (FF)-specific genes. (TIF 1210 kb) [file 12864_2018_4774_MOESM11_ESM.tif]

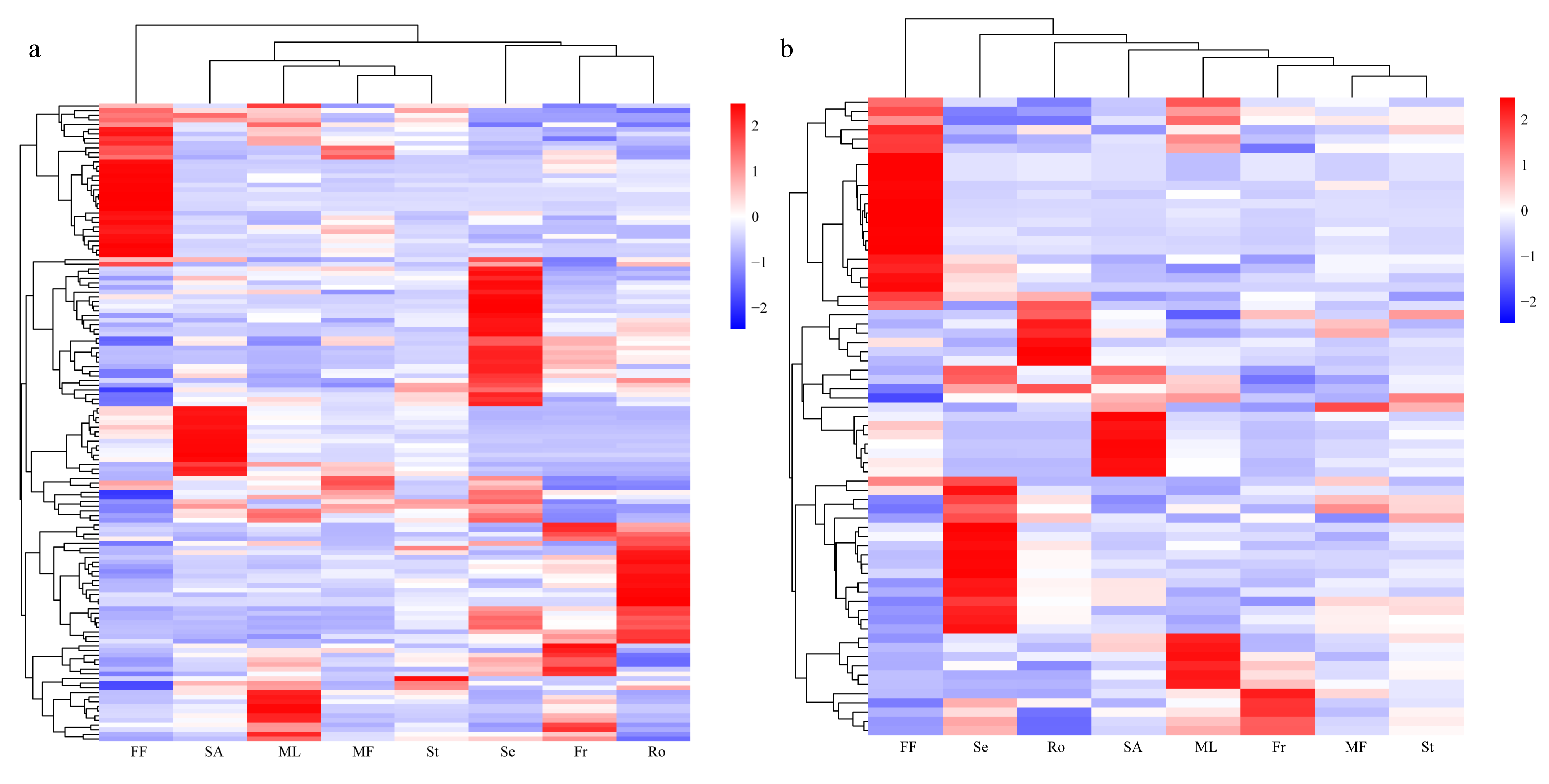

Supplement: Supplementary file 14 — Figure S8. Heat map representation and hierarchical clustering of putative genes involved in glycolysis/gluconeogenesis pathway and pentose phosphate pathway. A: glycolysis/gluconeogenesis pathway (ko00010). B: pentose phosphate pathway (ko00030). (TIF 2539 kb) [file 12864_2018_4774_MOESM14_ESM.tif]

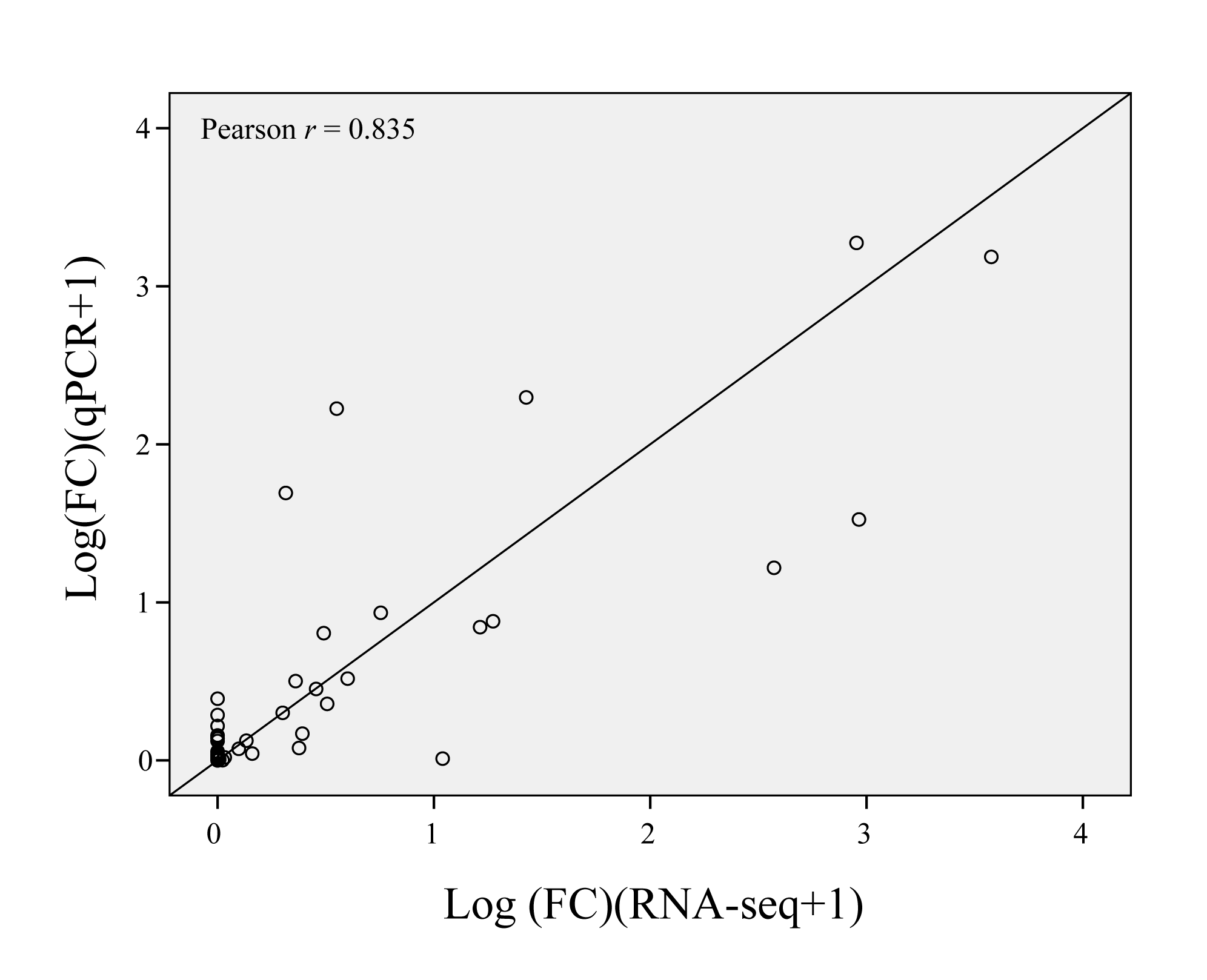

Supplement: Supplementary file 17 — Figure S9. Pearson correlation analysis of the gene expression ratios obtained from RNA-Seq and qPCR data. The qPCR log10 values (expression ratios; y-axis) were plotted against the RNA-Seq log10 values (x-axis). The Pearson correlation coefficient (r) is given in the plot, and the circle indicates the extremely significant difference at p < 0.01. (TIF 337 kb) [file 12864_2018_4774_MOESM17_ESM.tif]
